# Effect of various anticoagulants on the bioanalysis of drugs in rat blood: implication for pharmacokinetic studies of anticancer drugs

**DOI:** 10.1186/s40064-016-3770-4

**Published:** 2016-12-20

**Authors:** Preeti Kulkarni, Ashwin Karanam, Murari Gurjar, Sagar Dhoble, Arvind B. Naik, Bhaskar H. Vidhun, Vikram Gota

**Affiliations:** 1 Gahlot Institute of Pharmacy, Plot No 59, Sector 14, Koparkhairne, Navi Mumbai, Maharashtra India; 2 Department of Clinical Pharmacology, Advanced Centre for Treatment, Research and Education in Cancer (ACTREC), Tata Memorial Centre, Navi Mumbai, Maharashtra 410210 India

**Keywords:** Anticancer drugs, Bioanalysis, Plasma, Anticoagulant, Pharmacokinetics, Therapeutic drug monitoring

## Abstract

**Background:**

Pharmacokinetic studies are vital in development and optimization of drugs. While blood samples can be collected either in EDTA, heparin or citrate containing tubes for the estimation of drug levels in plasma, EDTA tubes are more commonly used. The purpose of this study was to evaluate the effects of anticoagulants on bioanalysis of drugs. Six drugs used extensively in cancer therapy were selected. Albino wistar rats (N = 6 per drug) were dosed with one of the following drugs intraperitoneally—pemetrexed (50 mg/kg), imatinib (50 mg/kg), erlotinib (25 mg/kg), meropenem (60 mg/kg), 6-mercaptopurine (20 mg/kg) and voriconazole (6 mg/kg). Blood samples were collected 2 h after dosing (1 h in 6-mercaptopurine group due to short half-life) by terminal bleeding from the retro-orbital plexus. Blood was collected in each of Disodium ETDA, heparin, trisodium citrate (TSC) and no anticoagulant (plain) tubes. Drug levels in these samples were determined by validated HPLC assays. ANOVA with Tukey’s post hoc test was performed to identify statistically significant differences in drug concentrations in anticoagulant tubes. p < 0.05 was considered statistically significant.

**Results:**

Significant differences in concentration between anticoagulant tubes was observed in case of erlotinib (p = 0.013) and meropenem (p = 0.00), while borderline statistical significance for pemetrexed (p = 0.076). TSC tubes overestimated erlotinib levels, heparin tubes underestimated meropenem concentrations and EDTA tubes overestimated pemetrexed concentrations.

**Conclusions:**

Careful selection of anti-coagulant is necessary for accurate characterization of pharmacokinetics of drugs. Routine use of EDTA tubes may lead to erroneous interpretation of pharmacokinetic data.

**Electronic supplementary material:**

The online version of this article (doi:10.1186/s40064-016-3770-4) contains supplementary material, which is available to authorized users.

## Background

Appropriate selection, sampling and storage of biological samples meant for estimation of drug levels is important, yet sometimes overlooked in day-to-day practice. Blood is one of the most important specimen of clinical interest as it provides unique advantages over other matrices in terms of wide variety of measurements possible, the vast amount of published data for both ante-mortem and postmortem drug level analysis, and the interpretive value of the matrix from a pharmacological standpoint. Blood samples can be collected in tubes having a wide range of options for preservatives, anticoagulants, and other additives and subsequently stored at room temperature, refrigerated, or frozen. The purpose obviously is to maintain the sample and the drug or its metabolites of interest in a state that will not degrade from the time of collection to the time of analysis. Needless to say drug testing labs have to develop methodologies for sample analysis that allows accurate estimation and interpretation of the data. For instance, interaction between drugs and the type of collection tube used is not uncommon and should be accounted for during analytical method development (Boeynaems et al. 2004; Smets et al. 2004; Wang et al. 2006).

Accurate determination of drug levels in human blood samples in clinical trials and routine patient care is extremely important. For instance, therapeutic drug monitoring (TDM) guides further dose selection of a drug based on levels achieved at previous doses. If doses are modified based on incorrectly reported drug levels, it could lead to sub-therapeutic levels or potential toxicity. For anticancer drugs and some anti-microbials with narrow therapeutic window and high toxicity, appropriate determination of analyte is vital for TDM (Chantry et al. 2014; Marquet and Rousseau 2008; Paci et al. 2014).

Ethylenediaminetertraaceticacid (EDTA), heparin, and trisodiumcitrate (TSC) are the commonly used anticoagulants. However, the effect of anticoagulants on the measurement of blood drug levels is not extensively reported in literature. We therefore conducted an experiment to investigate the effect of three commonly used anticoagulants, EDTA, heparin and citric acid, on plasma drug concentration measurements of six drugs commonly used drugs in cancer care.

## Methods

### Animals

Albino wistar rats (weight range 250 ± 20 gm) of either sex were used for the study. The experimental room was maintained under standard conditions of temperature (25 ± 2 °C) and relative humidity (55 ± 10%). Animals were subjected to 12:12 h light dark cycle. Animals were housed in standard polypropylene cages with wire mesh top and husk as bedding and allowed to acclimatize for one week before the start of the study. During this period, animals were fed with commercially available rodent food pellets and water ad libitum. The experimental protocol was approved by the Institutional Animal Ethics Committee (IAEC) of Gahlot Institute of Pharmacy and the experiments were carried out in accordance with the current guidelines for the care of laboratory animals.

### Experimental protocol

Six drugs commonly used in oncology practice i.e., 6-mercaptopurine (6-MP), meropenem, erlotinib, imatinib, voriconazole and pemetrexed were selected. Wistar rats (N = 36) were divided into six groups and each group was administered one of the listed drugs intraperitoneally at doses of 50 mg/kg for pemetrexed, 50 mg/kg for Imatinib, 25 mg/kg for erlotinib, 60 mg/kg for meropenem, 20 mg/kg of 6-MP and 6 mg/kg for Voriconazole. Blood was withdrawn from the retro orbital plexus after 2 h. In the case of 6-MP blood sample was collected 1 h after dosing owing to its short half-life. The dose and time point of collection was chosen based on literature evidence and reported half-life of these drugs in rats such that the drug concentration would fall within the linearity range of our HPLC assays (Pestieau et al. 2000; Wang et al. 2015; Hoshino-Yoshino et al. 2011; Harrison et al. 1989; Tterlikkis et al. 1977; Roffey et al. 2003). Blood sample from each animal was collected in three tubes containing different anticoagulants i.e. heparin, EDTA, TSC, and a fourth collection tube that did not contain any anticoagulant (plain tube). After collection, each collection tube was centrifuged at 3000 RPM at room temperature to separate plasma and was further analyzed for drug concentrations by HPLC.

### Chemicals

Acetonitrile (ACN), Methanol were procured from Sisco Research Laboratoty Pvt. Ltd, India. Dithiothreitol (DTT) and Perchloric Acid were obtained from Sigma Aldrich, USA. Dipotassium phosphate (K_2_HPO_4_), Formic acid and Orthophosphoric acid were procured from S D Fine-Chem Limited, India. Voriconazole was a kind gift of Pfizer, India. Erlotinib and Imatinib were kind gifts from Natco Pharma Limited, India. Pemetrexed was a kind gift of Dr. Reddy’s Laboratories. Meropenem and 6-Mercaptopurine were procured from Sigma Aldrich, USA.

### Sample preparation

The methods were developed in-house and validated according to the guidelines for validation of bioanalytical methods of FDA (Food and Drug Administration 2001). Validation was carried out for assay linearity, accuracy, precision and lower limit of quantitation (LLOQ). The coefficient of variation relative to reanalysis of the same sample was under 15% for all quality control samples of low, mid and high concentrations in the linearity range for all drugs. The following procedures were used for extracting drugs from the plasma:

#### Imatinib

1000 µL of ACN was added to 100 µL of plasma. This was vortexed for 5 min and then centrifuged at 15,000 RPM for 10 min at room temperature. 900 µL of the resulting supernatant was subsequently dried using LV Speedovap^®^ (Takahe Analytical Instruments) for 35 min at 40 °C. After complete drying, 100 µL of premix (ACN 80%, Water 20%) was added, vortexed for 5 min, and centrifuged at 15,000 RPM for 10 min. 90 µL of supernatant was transferred into autosampler vials and 30 µL was injected into the HPLC.

#### Erlotinib

1000 µL of ACN was added to 100 µL of plasma. This was vortexed for 5 min and then centrifuged at 15,000 RPM for 10 min at room temperature. 900 µL of the resulting supernatant was subsequently dried using Speedovap^®^ for 35 min at 40 °C. After complete drying, 100 µL of premix (ACN 75%, Water 25%) was added, vortexed for 5 min, and centrifuged at 15,000 RPM for 10 min. 90 µL of supernatant was transferred into autosampler vials and 30 µL was injected into the HPLC.

#### 6-Mercaptopurine

100 µL of plasma was mixed with 50 µL of DTT (75 mg/mL in water), 25 µL of water and 25 µL of perchloric acid. Vortexed for 5 min and subsequently centrifuged at 15,000 RPM for 10 min at room temperature. 150 µL of supernatant was kept in water bath (100 °C) for 45 min, after which it was cooled to room temperature and transferred into autosampler vials and 30 µL was injected into the HPLC.

#### Voriconazole

Briefly, 800 µL of ACN was added to 100 µL of plasma. Vortexed for 5 min and subsequently centrifuged at 15,000 RPM for 10 min at room temperature. 100 µL of supernatant was mixed with 100 µL Buffer solution (10 mM Ammonium Acetate, 5 pH), followed by vortexing for 2 min and then centrifugation at 15,000 RPM for 10 min. 100 µL of supernatant was transferred into autosampler vials and 50 µL was injected into the HPLC.

#### Meropenem

Solid Phase extraction was done using SPE cartridges. SPE cartridges were washed with 1 mL methanol followed by equilibration with 1 mL equilibration buffer (100 mM K_2_HPO_4_ pH 6.2). Plasma (100 µL) were then loaded into the cartridges. Secondary washing was performed with 500 µL of an equilibration buffer (pH 6.2). The samples were then eluted with 500 µL of 100% ACN followed by drying the eluent by Speedovap^®^ (40–45 °C). After drying, sample was reconstituted with premix (100 µL), vortexed for 2 min and centrifuged at 12,000 RPM. [Premix—A:B (5:95) where, A = ACN + Methanol (50:50), B = KH_2_PO_4_ Buffer pH 6.2]. 90 µL of the sample were loaded into HPLC vials and 30 µL was injected into the column.

#### Pemetrexed

Solid phase extraction was done using SPE cartridge. SPE cartridges were conditioned with 0.5 mL methanol followed by equilibration with 0.5 mL of 100 mM K_2_HPO_4_ (pH 6.2). Samples were then loaded in the cartridges. Secondary washing was done with 0.5 mL of 100 mM K_2_HPO_4_, pH 6.2 followed by 100% ACN (250 µL). Plasma (100 µL) was subsequently eluted with 0.5 mL of Formic acid: methanol (5:95). These were dried by Speedovap^®^ (40 °C) for 40 min. After drying, samples were reconstituted with 100 µL of 100 mM K_2_HPO_4_ (pH 6.2), vortexed for 5 min and centrifuged for 10 min at 15,000 RPM. 90 µL of this supernatant was loaded into HPLC vials and 30 µL was injected into the column.

### HPLC conditions

HPLC analysis was carried out under the conditions mentioned in Table 1. HPLC model: Dionex UHPLC Ultimate 3000 with diode array detector Dionex Ultimate 3000 RS was used. Kinetex^®^ (Phenomenex, USA) columns were used in the analysis.

**Table 1:** HPLC conditions maintained for analysis of study drugs

	Drugs					
	Erlotinib	Voriconazole	Imatinib	6-MP	Meropenem	Pemetrexed
Mobile phase	A:B = 40:60 A = acetonitrile B = ammonium acetate buffer (20 mM) pH 3.0	A:B = 35:65 A = acetonitrile B = ammonium dihydrogen phosphate buffer pH 7.0	A:B = 26.8:73.2 A = acetonitrile B = potassium dihydrogen phosphate buffer pH 3.0	A:B = 4:96 A = acetonitrile:methanol (50:50) B = triethylamine buffer pH 3.5	A:B = 5:95 A = acetonitrile:methanol (50:50) B = potassium dihydrogen phosphate buffer pH 6.2	A:B = 5:95 A = 1:1 methanol: ACN B = ammonium acetate buffer pH 6.2
Column	C18 4.6 × 100 mm, 5 μ	C18 4.6 × 100 mm, 5 μ	C18 4.6 × 100 mm, 5 μ	C18 4.6 × 100 mm, 5 μ	C18 4.6 × 100 mm, 5 μ	C18 4.6 × 100 mm, 5 μ
Column temperature (°C)	40	47	43	25	40	40
Flow rate (mL/min)	1	1	0.8	0.7	1	1
Retention time (min)	4.2 + 0.5	5.7 + 0.5	3.4 + 0.5	3.7 + 0.5	6.67 + 0.5	6.7 + 0.5
UV detection wavelength (nm)	247	255	265	324	297	255

### Statistical analysis

All statistical tests were performed using SPSS 20 (IBM). The samples collected in plain tubes (without any anticoagulant) were considered as control group and the concentrations observed in anticoagulant tubes were normalized against this value. We define ‘plain normalization’ as ratio of concentration of the drugs in each of the anticoagulant tubes with corresponding concentrations in plain tubes of the same animal. This normalization was carried out in order to eliminate interanimal variability in pharmacokinetics, thus making it possible to attribute differences in plain normalized values between anticoagulant groups to the anticoagulant effect. ANOVA, with Tukey’s post hoc test was used to compare the plain normalized values between the anticoagulant groups. A p value of less than 0.05 was considered statistically significant.

## Results and discussion

**Fig. 1:**
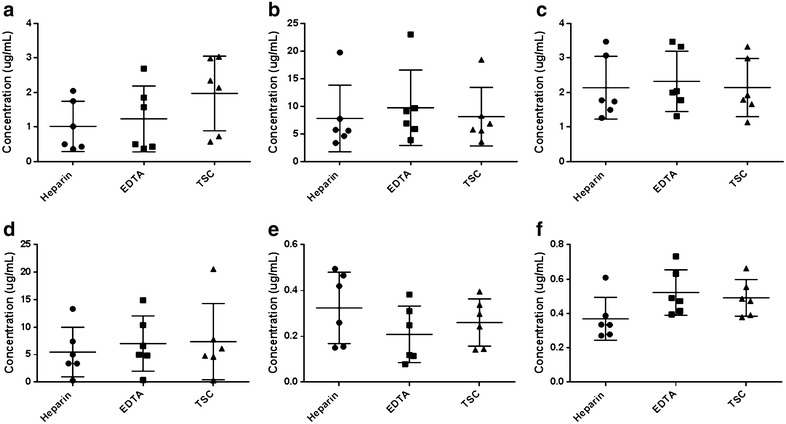
Individual plasma concentrations of **a** erlotinib, **b** pemetrexed, **c** voriconazole, **d** imatinib, **e** 6-mercaptopurine and **f** meropenem when collected in heparin coated, EDTA coated and TSC coated tubes

**Fig. 2:**
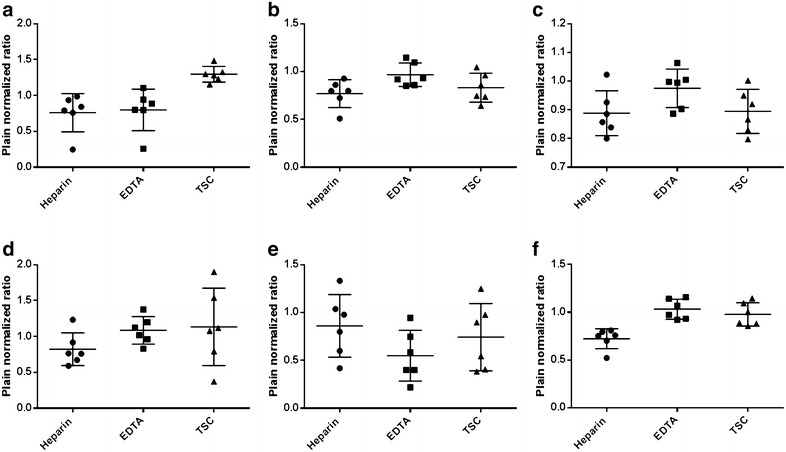
Individual plain normalized ratios of **a** erlotinib, **b** pemetrexed, **c** voriconazole, **d** imatinib, **e** 6-mercaptopurine and **f** meropenem when collected in heparin coated, EDTA coated and TSC coated tubes

**Fig. 3:**
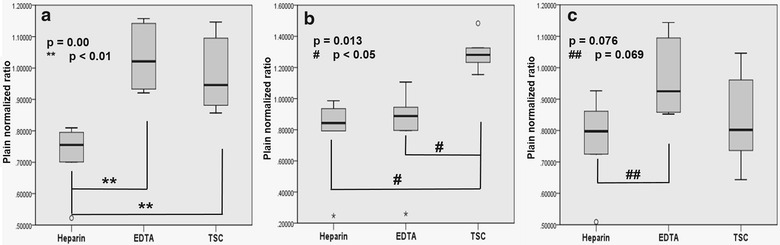
*Box* and *whisker* plots of plain normalized ratios of **a** pemetrexed, **b** meropenem, **c** erlotinib depicting statistical significance at indicated p values

Validation parameters including linearity, accuracy, precision and LLOQ for each analyte is shown in Table 2. The concentration of drugs determined using different anticoagulants is presented in Fig. 1 (The raw data showing concentration of each drug observed in each animal for different anticoagulants may be found in Additional file 1). As seen from the figure, we observed significant inter-animal variability within the same anticoagulant group. The plain normalized ratios are presented in Fig. 2, depicting a tighter set of observations by eliminating interanimal variability, and thereby bringing anticoagulant effect to the fore as in the case of erlotinib and meropenem. ANOVA test revealed statistically significant differences in concentration in case of erlotinib (p = 0.013) and meropenem (p < 0.01) while borderline statistical significance for pemetrexed (p = 0.076) between various anticoagulants as seen in Fig. 3. Between group comparison using Tukey’s post hock test showed that heparin tubes significantly underestimated meropenem concentrations when compared to EDTA (Tukey, p = 0.001) and TSC tubes (Tukey, p = 0.003) (Fig. 3a). The significance of this observation lies in the fact that meropenem is a time-dependent antibiotic which exerts antibacterial activity more efficiently if the plasma concentration is more than the Minimum Inhibitory Concentration (MIC) of the targeted strain of bacteria for at least 40% of the dosing interval (fT > MIC > 40%) (Drusano 2003). Under such circumstances, using heparin tubes for blood collection may grossly underestimate the fraction of time above MIC (fT > MIC) resulting in unnecessary dose modifications. In case of erlotinib, TSC tubes significantly overestimated the concentration of the drug in comparison to heparin (Tukey, p = 0.019) and EDTA tubes (Tukey, p = 0.028) (Fig. 3b). In case of pemetrexed, concentrations in the EDTA tubes tended to be higher than heparin tubes but the difference was not statistically significant (Tukey, p = 0.069) (Fig. 3c). Anticoagulant effect was not observed in the case of voriconazole, imatinib and 6-MP.

**Table 2:** Validation parameters of linearity, accuracy, precision and LLOQ for each analyte

Analyte	Linearity range (μg/mL)	Accuracy*	Precision* (CV %)	LLOQ (μg/mL)
Erlotinib	0.1–10	95.6–98.7	3.7–10.5	0.1
Pemetrexed	0.1–10	96.2–101.1	2.9–12.1	0.1
Voriconazole	0.25–8	100.4–102.3	6.0–9.7	0.25
Imatinib	0.1–10	103.3–101.7	3.4–5.7	0.1
6MP	0.1–10	102.4–107.4	4.6–6.1	0.1
Meropenem	0.1–10	89.5–97.2	4.3–4.5	0.1

*CV* coefficient of variation, *LLOQ* lower limit of quantitation

* Range of accuracy and precision values shown for low, medium and high QC samples

Commonly used anticoagulants for blood collection include EDTA, heparin and citrate, EDTA being used most frequently. However, EDTA is a known chelator and hence not used in estimation of metal ions. Cisplatin for example, is collected in heparin coated tubes. Identification of such interactions is key to minimizing influence of any external factors on the analyte concentrations. Barring a few examples, mechanisms by which anticoagulants affect the measurement of drug concentrations is poorly understood. A study similar to ours was performed by Chen et al. (2008) with tigecyclin and ciprofloxacin in rats, using EDTA and heparin collection tubes. They concluded that EDTA might compete with tigecyclin and ciprofloxacin for chelating metal ions, thereby affecting drug partitioning in the plasma compartment leading to inaccurate estimation of drug levels.

Accurate detection of analyte concentrations is one of the most important challenges faced in clinical setting. The FDA Guidance to Industry for bioanalytical method validation necessitates evaluation of matrix effect (including anticoagulants) before the method can be adopted for bioanalysis of drugs, however there are no guidelines pertaining to how these effects should be evaluated Food and Drug Administration (2001). This is often overlooked in non-regulatory studies which constitute the major segment of published literature, thereby perpetuating errors in the estimation of pharmacokinetic parameters of drugs. Our findings provide compelling argument in favor of identifying the compatibility of anticoagulants with the analytes. To the best of our knowledge, this is the first study which has systematically evaluated anticoagulant effects on the bioanalysis of drugs used in cancer care. Since some of these drugs are good candidates for pharmacokinetics guided dose optimization or TDM (Moriyama et al. 2015; De Keukeleire et al. 2016), and there is evidence emerging in case of some others (Herviou et al. 2016), the findings of this study clearly underscore the importance of choosing the right anticoagulant for pharmacokinetic studies and bioanalysis of TDM samples. The findings are also relevant to other fields including forensic medicine where plasma drug levels are often analyzed.

The importance of type of anticoagulant coated collection tube does not only apply for drugs and metabolite concentrations, but has to be extended to other biological analytes as well. The influence of various anticoagulants in biochemical analytes have been substantiated by several studies. Heparin has been identified to interfere with estimation of thyroid hormones and albumin levels (Bowen and Remaley 2014). Chuang et al. (1998) described the influence of anticoagulants in amino acid analysis. They identified that EDTA reacts with ninhydrin reagent used in amino acid analysis and produces a ninhydrin positive contaminant, which interferes with the analysis. Similarly, they suggested that heparin coated tubes may interfere with estimation of sulfur containing amino acids due to presence of a preservative, sodium metabisulfite. Suttnar et al. (2001) demonstrated the underestimation of malonaldehyde levels (a marker of lipid peroxidation), when collected in EDTA tubes. Lykkesfeldt (2012) noted that use of EDTA tubes prevented ex vivo oxidation of oxidative stress biomarkers ascorbate and dehydroascorbic acid when compared to 5 other anticoagulants. Mohri and Rezapoor (2009) also demonstrated the difference in estimating routine biochemical parameters using EDTA, heparin and citrate tubes. The chelating nature of EDTA and its influence on samples collected for blood clotting assay and serum electrolytes was shown by Lima-Oliveria et al. (2014, 2015). Cross-contamination of heparinized or citrated blood with EDTA was shown to adversely affect the blood clotting assay and also the electrolyte levels, grossly underestimating them. A study by Wiese et al. (1997) recommended the use of EDTA or heparin tubes over citrate tubes as they led to lower lactate concentration measurement in critically ill.

The spectrum of influence of anticoagulants on analytes is not only limited to interference with their concentrations, but also in analyte stability. More often than not, blood samples are not processed immediately. Rather, they are refrigerated and are then analyzed at a later point of time. This was elucidated by Lam et al. (2004) for DNA analysis, wherein they suggested use of EDTA as anticoagulant of choice for delayed blood processing.

Some limitations of our study include a small sample size leading to lack of statistical power. However, the study still manages to establish the proof of concept in spite of this limitation. Secondly, we did not use internal standards (IS) in our HPLC assays since we did not want to confound our observations by introducing another factor (the IS) which may, on its own, be influenced by the anticoagulant effect. Instead, we relied on robust validation of the assays as per FDA bioanalytical validation guidelines.

## Conclusions

To conclude, the choice of anticoagulant influenced the estimation of plasma concentration for three out of 6 drugs studied. Influence of anticoagulants on the estimation of drug levels should be a guiding factor in choosing an appropriate anticoagulant for accurate characterization of pharmacokinetics of drugs. EDTA as the anticoagulant of choice is questionable and may lead to erroneous interpretation of pharmacokinetic data in some cases.

## Additional file

**Additional file 1.** Raw data showing concentration observed for each drug in each animal following collection in EDTA, heparin, TSC and plain tubes.

## Abbreviations

TSC: trisodium citrate
EDTA: ethylenediaminetertraaceticacid
IAEC: Institutional Animal Ethics Committee
6-MP: 6-mercaptopurine
ACN: acetonitrile
DTT: dithiothreitol
LLOQ: lower limit of quantitation
MIC: minimum inhibitory concentration
fT > MIC: fraction of time above MIC
TDM: therapeutic drug monitoring
